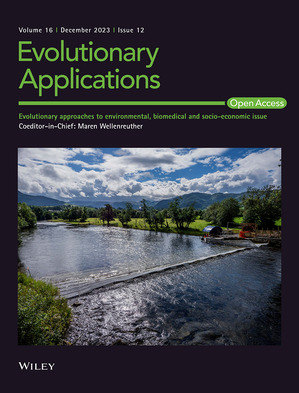# Cover Image

**DOI:** 10.1111/eva.13415

**Published:** 2023-12-21

## Abstract

Caption: The migration trap at Havforskningsinstituttet's field platform on the river Etne permits sampling of almost all adult Atlantic salmon entering the river to spawn.

Credit: Øystein Paulsen, HI.